# Prescriber Attitudes Towards Prescribing 'Green' Inhalers in Secondary Care

**DOI:** 10.7759/cureus.88986

**Published:** 2025-07-29

**Authors:** Aazaz Rehman, Joseph Marchant, Jiakun Yu, Holly Hathaway, Matt Pavitt, Sabrine Hippolyte, Ketan Patel

**Affiliations:** 1 Respiratory Medicine, University Hospitals Sussex, Brighton, GBR; 2 General Medicine, University Hospitals Sussex, Brighton, GBR; 3 Trauma and Orthopaedics, Worthing Hospital, Worthing, GBR; 4 Respiratory Medicine, Brighton and Sussex Medical School, Brighton, GBR; 5 Respiratory Medicine, Barts Health NHS Trust, London, GBR

**Keywords:** airways disease, environmental sustainability, inhaler therapy, inhaler use, mixed-method approach, prescribing behaviour, qualitative research

## Abstract

Introduction

The climate emergency is advancing at a rapid rate and affecting every corner of the globe. Healthcare has been calculated to be responsible for a considerable part of global greenhouse gas (GHG) emissions. Dry powder inhalers (DPIs) are a lower-carbon alternative to pressurised metered dose inhalers (MDIs); however, many patients remain on MDIs. This study aimed to identify barriers to switching to DPIs, as a trust-wide audit of pressurised MDIs (pMDIs) revealed that very few of the patients eligible for a DPI were offered it in secondary care, despite sustainability initiatives.

Methods

We conducted interviews focusing on the perceptions and experiences of secondary care healthcare professionals (HCPs) relating to inhaler prescribing. Potential participants were selected through purposive sampling to ensure a broad perspective. Semi-structured interviews were conducted, and their transcripts were coded using QSR NVivo (Burlington, MA). Qualitative data were organised and analysed thematically using the framework method.

Results

Nineteen participants were recruited from varying grades and disciplines within secondary care. Three themes relating to attitudes towards sustainable inhaler prescribing emerged: 1) the awareness of suitable inhalers, 2) knowledge and 3) making an impact.

Conclusion

HCPs indicated a desire to reduce the carbon footprint generated by healthcare. However, the poor awareness of the inhaler carbon footprint acted as a barrier to promote sustainable inhaler prescribing. Where participants were aware of their carbon footprint, a lack of knowledge and confidence regarding inhalers prevented a change to sustainable inhalers. Overall, this study shows that existing sustainability initiatives are not delivering the intended impact and that more work is required around raising awareness and education regarding inhalers.

## Introduction

The world is in a state of climate emergency; despite this, over 35 billion metric tonnes of carbon dioxide (CO_2_) was released into the atmosphere in 2023 [1], and this continues to increase every year with 'no sign of slowing down' [2]. The climate change emergency has been shown to affect the poorest countries and the most vulnerable people across the globe [3]. In 2014, the healthcare sector was calculated to be responsible for 4.4% of the total global greenhouse gas (GHG) emissions [4]. If the sector was a country, it would have the fifth highest carbon footprint on Earth [5]. In the United Kingdom, asthma affects almost 5.4 million people, and chronic obstructive pulmonary disease (COPD) affects five million people, costing the NHS five billion pounds annually [6-8]. Hospital admissions for respiratory disease are rising at three times the rate of all admissions, with incidence and mortality rates higher in disadvantaged groups [8]. Due to rising temperatures in the United Kingdom, common pollens such as oak and grass have an earlier and longer onset [9]. Increased temperatures and heatwaves lead to worse air quality [10]. Higher pollen and worse air quality then lead to increased asthma-related healthcare visits and hence a higher carbon footprint, forming a vicious cycle [11].

The NHS has set a target to reduce its carbon footprint by 80% by 2028-2032 [12]. Three percent of the total NHS carbon footprint is due to inhalers, of which the majority are from pressurised metered dose inhalers (MDIs) [12]. An alternate, lower-carbon option is dry powder inhalers (DPIs). A typical MDI inhaler emits 28-35 kg of CO_2_, which is equivalent to a 115-mile car journey, whereas a typical DPI emits less than 1 kg of CO_2_ [13]. In Europe, and particularly in the United Kingdom, individuals with asthma over-use short-acting beta-agonist (SABA) inhalers and should be escalated in terms of their 'preventer' management [14]. One of the major causes of greenhouse gas (GHG) burden from inhalers is through pressurised MDI (pMDI) SABA inhalers, the use of which has been associated with increased risk of exacerbations [14]. Nearly 47% of individuals in the United Kingdom with asthma have poorly controlled asthma. These individuals also have higher healthcare visits, causing an eightfold higher contribution to GHG emissions than those with well-controlled asthma [15].

The complex issue around inhalers requires a multi-faceted approach, including improving diagnosis and education to facilitate switching to DPIs. Firstly, patients must be diagnosed correctly with asthma or COPD. Those individuals with a confirmed diagnosis, in the first instance, should be counselled and educated about the use of DPI devices. However, in the United Kingdom, approximately 70% of inhalers prescribed are pMDIs compared to less than 30% in European countries [16]. SABA inhaler prescription demand must be reduced through the prompt escalation of treatment for individuals with asthma who are using more than three SABA containers within 12 months; this is reflected in the new British Thoracic Society (BTS)/Scottish Intercollegiate Guidelines Network (SIGN)/National Institute for Health and Care Excellence (NICE) guidance where individuals with new diagnosis of asthma are to be initiated on inhaled corticosteroid (ICS)/long-acting β2 agonist (LABA) inhalers as first-line therapy. For those patients who are already on pMDIs, where suitable based on patient factors, we must switch them to corresponding DPIs. DPIs decrease the carbon footprint with no loss of asthma control despite concerns [17].

Whilst it may be thought that inhaler initiation and switching are primary care issues, 42% of patients miss their yearly asthma reviews, and 38% of patients miss their annual COPD review [18,19]. When these patients present to secondary care, there is an excellent opportunity to perform a review of their airway disease and optimise their inhalers by healthcare professionals. Not only can inhalers be optimised by escalating patients up the treatment ladder, but also patients can also be counselled to use lower-carbon footprint inhalers.

## Materials and methods

This project was registered as a local quality improvement project (QIP) and utilised a sequential, mixed-methods explanatory approach. Initially, a prospective audit of current practice in our trust across two hospital sites was conducted from 1 to 30 November 2023 across two acute hospitals within the University Hospitals (UH) Sussex Foundation Trust. The aim was to determine whether patients were being switched to DPI-equivalent inhalers. Patient demographics and indication for inhaler use were checked through electronic general practitioner (GP) records and hospital letters. Patient discharge summaries were accessed from 1 December 2023 to 1 March 2024. Data regarding changes to the regular inhaler were collected from inpatient episode discharge summaries.

Based on the findings of the audit, a key question emerged regarding the lack of offering dry powder inhalers (DPIs) to patients within secondary care. To address this, we conducted qualitative research through semi-structured interviews utilising a topic guide (see Appendices), which emphasised exploring participants' perceptions and lived experiences concerning the prescribing and switching to 'green' inhalers. The aim was to understand the barriers to sustainable inhaler prescribing within an acute hospital setting.

Patient selection

All patients admitted on an inhaler were collated via e-prescribing and discharge data. All patients admitted to an inpatient medical bed and over the age of 16 at the time of admission and successfully discharged from the hospital were included.

Participant selection

The qualitative study enrolled resident doctors of all grades and across all medical specialties, as well as pharmacists, through purposive sampling. The participants all worked at University Hospitals Sussex across Brighton and Haywards Heath. The participant cohort did not include any respiratory trainees, as this could have led to bias in knowledge. Consultants were not included in the participant cohort primarily because their prescribing responsibilities in an inpatient setting are less compared to resident doctors. Whilst consultants oversee patient care through critical decision-making, the practical aspects of prescribing, such as initiating medications, adjusting doses and completing discharge paperwork, are typically handled by resident doctors. By focusing on resident doctors, the study targets those directly responsible for daily prescribing decisions, ensuring the findings reflect the real-world challenges and habits of inpatient prescribing.

A target sample size was not predetermined in this case, and interviews were conducted with the aim to achieve theoretical data saturation. Although there are many factors that can influence saturation, we ceased data collection when no new viewpoints emerged from the interviews.

Data collection

The interviews were conducted between 16 May 2024 and 17 December 2024 by AR (lead researcher) and JY (clinical researcher), both resident doctors working in the hospital trust at the time. Both had received previous education on qualitative research methodology. Resident doctors and pharmacists were identified by AR and JY and then approached for participation. All participants were given a priming leaflet prior to their interviews.

The interviews were conducted in a semi-structured manner with a topic guide created by AR, JY and KP in advance (see Appendices). The interviews were conducted either face-to-face or via Microsoft Teams (Microsoft Corp., Redmond, WA), depending on participant preference. Written and verbal consent was obtained from all participants. The participants were aware that AR and JY were resident doctors and KP was a respiratory physician. The interviews were audio-recorded and transcribed. No repeat interviews were carried out.

Data analysis

Interview transcripts were analysed using a framework method for qualitative data analysis [20]. Transcripts were coded using QSR NVIVO (Burlington, MA) to aid with quote extraction during the creation of the matrix framework. Three transcripts deemed to be informative were coded by the researchers. The coded transcripts were discussed in a workshop to agree on initial codes. The process was repeated once more with three further transcripts to agree upon the initial coding framework. This initial framework was then used by AR and JY to code all remaining transcripts, and any new codes generated were then discussed by AR, JY and KP as they emerged. Once the matrix framework was created, similar codes were combined to create subthemes and themes that answered the research question.

Ethical approval

NHS Research Ethics Committee (REC) approval was not required as per the Health Research Authority (HRA) recommendation. The project was registered within the trust as a service improvement project and was subject to trust governance protocols (project ID 2106). The UK Research and Innovation (UKRI) Medical Research Council Health Research Authority issued approval 340699.

## Results

We identified a total of 143 patients, of whom 101 were on an MDI that could be switched to a lower-carbon footprint inhaler. Ten patients had no diagnosis or spirometry explaining inhaler use. Six percent of patients on a preventer MDI were offered a DPI alternative. No patients were offered a DPI alternative for salbutamol despite 21 patients already being on a DPI preventer inhaler. The data are summarised in Table 1.

**Table 1:** Audit data on inhaler prescribing MDI, metered dose inhaler; DPI, dry powder inhaler; COPD, chronic obstructive pulmonary disease

Diagnosis	n	On preventer MDI	On preventer DPI	Preventer DPI offered (percent of MDIs)	Salbutamol MDI	Salbutamol DPI	Salbutamol DPI offered
Asthma	51	36 (71%)	9 (18%)	1 (3%)	49 (96%)	2 (4%)	0
COPD	81	62 (76%)	15 (19%)	5 (8%)	78 (96%)	1 (2%)	0
No diagnosis	10	3 (30%)	0	0	10 (100%)	-	0
Mixed airway disease	1	0	1 (100%)	0	1 (100%)	0	0
Total	143	101 (71%)	25 (17%)	6 (6%)	138 (97%)	4 (3%)	0

Participant characteristics

Thirty participants were contacted, of whom 19 agreed to an interview. The participants were medical residents working on the acute medical take and prescribing pharmacists in the trust who were identified by the respiratory lead pharmacist. The participants who did not participate were unable to due to time pressures. The average interview time was 22 minutes. Participant demographics are shown in Table 2.

**Table 2:** Summary of participant demographics FY, foundation year; IMT, internal medicine trainee

Participant role	Number of participants	Number of male participants	Number of female participants
FY1	5	3	2
FY2	5	2	3
IMT	3	1	2
Specialty trainee	4	2	2
Pharmacist	2	0	2

Thematic analysis

The transcripts of the participants were analysed and divided into three main themes and 17 subthemes. These represented the largest barriers to switching to green inhalers from an acute secondary care perspective. These were determined using the codes described in Appendices. A summary of these themes is shown in Table 3.

**Table 3:** Inhaler themes and quotes from data analysis MDT, multidisciplinary team; EPMA, Electronic Prescribing and Medicines Administration; VTE, venous thromboembolism

Themes	Subthemes	Codes	Quotes
Awareness of sustainable inhalers	Awareness of inhaler footprint	Inhaler knowledge. Inhaler education. Impact of green intervention. Guidelines. Green inhaler awareness. Green awareness initiative. Green agenda awareness	'If I had the appropriate training and clinical backing behind me to be able to make that decision, I would. If it was as simple as switching the same drug to a different device that's more green, then I think I'd be quite happy doing that' - 014. 'So, I think I know the drug names, and I know broadly how they work, but I know nothing about the environmental impact, and I know very little about the practicalities of actually taking the drug' - 022.
	Lack of teaching on sustainability	Education about green agenda. Green inhaler awareness. Inhaler education. Inhaler knowledge	'I mean, it's not something that was covered very much at uni. It was, you know, you, you learn the ladder for someone with asthma or something like that, but you don't learn the practicalities of different devices and different, um, especially not sort of environmental consequences of inhalers' - 006. 'It's just a simple concept. Like if you can choose an environmentally friendly inhaler, you should choose an environmentally friendly inhaler. I don't think we got much teaching about inhalers in medical school as well' - 021.
	Current culture in the NHS is not aimed at reducing carbon footprint	Impact of green intervention. Health policy. Healthcare culture. Guidelines	'I guess, yeah, I guess the focus that we get, all the teaching and stuff that comes to us is more about advances in treatment or new guidelines and how to change your clinical practice. And there's not really an emphasis at all about the impact of your care on the environment. And certainly, like just day-to-day, it seems very wasteful, a lot of the things we do, like we have a lot of single-use things, some of them for good reason, but often just the like the amount of packaging, for example, for things seems excessive' - 003.
	Interest in a green agenda	Green inhaler awareness. Guidelines. Green agenda awareness	'Yeah, I mean I don't know I hope this is true, but I don't know that this is true that I hope most people are fairly interested in the environment, and it's not it's no skin off my nose if I prescribe a different type of inhaler; it's basically I don't have to do anything I just have to write slightly different letters; yeah that's like minimal effort to like do your little part for the environment, so there really shouldn't be too much pushback or wouldn't have thought, and it's partly a generational thing. I think we are a bit more environmental conscious from the certainly the people I know' - 022.
	Lack of confidence to make changes to inhaler regimes	Barrier to intervention. Inhaler prescribing behaviour. Inhaler knowledge. Inhaler education. Inhaler confidence. Healthcare culture. Changing inhalers	'And I definitely don't have an understanding to switch them to something more environmentally friendly. I would switch someone from, if they came in on a salbutamol inhaler, I would put them onto a salbutamol nebuliser, but that's about as much as I would do. I wouldn't want to change people's inhalers' - 006. 'I think I would feel comfortable suggesting it. But again, it goes back down to knowledge. So, if I did have more knowledge about it, I would feel more confident because then if you get asked why, you'd be able to explain it a bit better' - 016.
Confidence with inhaler prescribing	Timing of inhaler switching	Prioritisation. MDT involvement. Inhaler prescribing behaviour. Changing inhalers	'I think the main barrier is just the essentially knowledge of when it's okay to switch and when you, you know, just my knowledge of when you can and can't switch devices. That's my main barrier' - 018.
	Senior-led decision-making	Changing inhalers. Barriers to intervention. Inhaler prescribing behaviour. Inhaler confidence. Healthcare culture. Changing inhalers	'I think if I presented with the situation I wouldn't feel particularly empowered to make that decision. I think that I would assume that I would have to get someone else's opinion before I'd be able to order out to make decision. I don't know whether that's legitimate or not, but I know that that's something that I guess I subconsciously would think' -020. 'I think if knowing that seniors are happy for us to make those decisions, that would be good because I know, I know some consultants would be like fine change their inhalers; they wouldn't. They wouldn't mind too much. But some will be like, "why did you do that without checking?" You know, like it depends on who you're with' - 005.
	Cost and availability of medications	Barrier to intervention. MDT involvement. Inhaler prescribing behaviour. Healthcare culture. Green agenda awareness. Changing inhalers	'So, I think, yeah, training, knowledge, awareness are definitely all barriers and potentially cost and availability as well. If they're more expensive, then our trust might not necessarily want to help facilitate that' - 014.
	Reliance on pharmacists or nurses for guidance	Opportunity. MDT involvement. Inhaler prescribing behaviour. Inhaler knowledge. Inhaler confidence. Green inhaler awareness	'Okay yeah, I think perhaps pharmacy would probably be the best place to start because if pharmacy just stopped, then I'm assuming these are equally clinically effective. Yes. If pharmacy just stopped the green ones, then it would sort of become, you'd have to prescribe the one pharmacy stock isn't it' - 022. 'If someone told me what I mean, I don't know, I wouldn't know what to change it to at the moment. But if someone told me what I needed to change it to, then I probably I would do it' - 004.
	Guidelines are the most helpful tool	Modifying prescribing behaviour. Interventions. Inhaler prescribing behaviour. Guidelines	'That would be so helpful. I think we rely on microguide for so much, and especially as juniors when we're unsure; that's the first kind of point of accessing information. So, if we had clear guidelines or recommendations there, it would be very useful to start initiating those changes' - 016. 'I guess one thing that I think of is just more sort of freely accessible guidelines and clear guidelines on when you can change and in what situations, you know, a foundation doctor would be able to change something or in what situations you'd need specialist input' - 018. 'That's a really good idea yeah. I like that you could have an e-prescribing warning box that says do you know there is a green alternative sort of thing. I'd probably try and make it a different colour to the other warning boxes that come up because you get so many you get used to just clicking through. But yeah, that would be a really good plan' - 022.
Making an impact	Widespread disseminated information is not a useful way to educate	Learning	'Vast majority of people that have these weekly news roundups or information of the week or whatever probably about 10% of people that I've seen or less actually read it; yeah, I probably read maybe one in three, and I would count myself in that 10%' - 024.
	Small group, simulation and case-based education are the preferred methods of learning	Learning. Interventions. Education modality. Education delivery	'So, I would think that having it in a grand rounds type format as a, these are the data, this is the science behind it, these are the cost savings, this is the environmental impacts, why are we not doing this, this is what we should be doing, is entirely reasonable at registrar and consultant level. But I think trickle down, because juniors are, the bandwidth of many juniors is so overwhelmed by stuff; they don't already know they're absorbing at an accelerated rate, but taking that on board as well is quite hard in a formal setting' - 024. 'A teaching program to teach doctors about sustainability in medicine in general? So, I suppose like if that was in the form of like some sort of like a presentation or something if that's what you're talking about I guess I would try and start off by introducing them to the topic of you know how the carbon footprint of the NHS and where the biggest sort of the least eco-friendly' - 020.
	Incorporating green prescribing education early in a person's training	Opportunity. Interventions. Inhaler education. Education delivery	'Um, foundation, I suppose, I suppose that foundation is still a point of interest and I think they probably should be having, I imagine some respiratory teaching as part of foundation. So, I think it could be done as part of that. Um, because they also might be useful and even if they're not making the decision to switch to raising that to the ward team, should this be switched possibly?' - 003.
	Posters are useful reminders	Learning. Interventions. Education modality. Education delivery	'The right balance of colours so it's got to capture your attention without being overwhelming; it's got to have arrows right; arrows help to guide your next thought to the end of the poster, so it seems brief we've made absorbed a fair bit of information, so I think that's important um having it so that it doesn't seem as a balance between not patronising not dictatorial but yet informative to people know and that's quite a difficult balance to strike' - 024.
	The benefit of prompts when using electronic prescribing	Opportunity. Modifying prescribing behaviour. Interventions. Inhaler prescribing behaviour	'Or even circling back to EPMA again, you know how the first thing I can think of is when you prescribe VTE, it comes up with like this dose for this prescription this way. You can have something on that first tab when you click the medicine that says, there is this and this greener version of this inhaler that has the same efficacy. And you can prescribe that as follows. And that would kind of be a little note that would then educate and maybe link you to more research. While also prompting you to then switch to the other inhaler' - 017. 'On EPMA, you know, obviously on the prescribing bit like keeping it quite simple, is probably better, because not many people go through read all the patient all the notes bits, you know, and if it ended up in there, it probably would get missed. But I think like a little icon that then a little something that you could then click, and it links it to the carbon footprint information could be quite good' - 008.

Theme 1: Awareness of sustainable inhalers

It was perceived that due to poor teaching from university level upwards, the participants' understanding of inhalers and their environmental impact was poor and therefore a large barrier to them switching a patient's inhalers.

I mean, it's not something that was covered very much at uni. It was, you know, you, you learn the ladder for someone with asthma or something like that, but you don't learn the practicalities of different devices and different, um, especially not sort of environmental consequences of inhalers. - Participant 006

It was perceived that the 'green agenda' within the NHS was not something widely discussed or prioritised. The participants highlighted that teaching concentrated on the clinical impact of inhalers and rarely included the environmental impact of these choices. Most participants, however, did have an interest in understanding and improving the environmental impact of their actions. It was perceived that the new generation of prescribers cared about reducing their carbon footprint and had an understanding that their actions did have an impact on the climate crisis.

I guess, yeah, I guess the focus that we get, all the teaching and stuff that comes to us is more about advances in treatment or new guidelines and how to change your clinical practice. And there's not really an emphasis at all about the impact of your care on the environment. And certainly, like just day-to-day, it seems very wasteful, a lot of the things we do, like we have a lot of single-use things, some of them for good reason, but often just the like the amount of packaging, for example, for things seems excessive. - Participant 003

Theme 2: Confidence with inhaler prescribing

The lack of confidence in inhaler prescribing was a consistent theme from all participants. It was felt that support from consultants, pharmacists and clinical nurse specialists was important. This perceived lack of confidence appeared multifactorial; however, two subthemes that were identified were 'concern about going against the respiratory team's advice' and a 'lack of clear guidelines'.

Yeah, I'd, I'd feel more worried that I would like annoy that person's usual respiratory team if I had changed their inhaler. - Participant 004

I guess one thing that I think of is just more sort of freely accessible guidelines and clear guidelines on when you can change and in what situations, you know, a foundation doctor would be able to change something or in what situations you'd need specialist input. - Participant 018

An understanding about the variety of inhaled devices plus uncertainty regarding the best time to switch to green inhalers were consistently highlighted as a barrier. Having the knowledge of the inhaler types, subsequent medications and devices stopped participants from initiating switching. This often led to the participants saying that they would need senior support in initiating or changing inhalers. Many also mentioned the high clinical demand on resident doctors, meaning switching patients' inhalers was not their clinical priority. This concentration on acute illness was also compounded by the participants not wanting to impact on their underlying respiratory illness control, with an uncertainty that switching inhalers during an acute admission was the most appropriate time.

Yeah, I don't have the priority capacity, but then also if I was going to look at someone's inhalers, I would want to do a proper review of their, how they use their inhaler, and A&E is just often not the right place to do that, often because they haven't got their inhaler with them and often when they do come in if they're there with reasons to do with their respiratory system they're normally put on nebulisers or in an acute exacerbation where trying to get anyone to show me their inhaler technique is pretty impossible; yeah, so I think it's just not the focus of my mind, and obviously when I'm on call, I don't think I think about it at all. - Participant 007

Theme 3: Making an impact

A large barrier to positive change was the inability to effectively disseminate information about sustainable inhalers to resident doctors and prescribers. Mass emails and large group teaching were thought to be an ineffective way to pass on information. One perceived area where impactful change could occur was the inclusion of prompts on the electronic prescribing system ('EPMA', Electronic Prescribing and Medicines Administration).

You can have something on that first tab when you click the medicine that says, there is this and this greener version of this inhaler that has the same efficacy. And you can prescribe that as follows. And that would kind of be a little note that would then educate and maybe link you to more research. While also prompting you to then switch to the other inhaler. - Participant 017

Small group teaching, as well as on-the-ward teaching from senior colleagues, was thought of as effective methods of teaching. Some believed that the responsibility should lie with more senior doctors to decide upon these inhaler switches and that the cognitive load of more junior resident doctors was too high to consider these changes.

So, I would think that having it in a grand rounds type format as a, these are the data, this is the science behind it, these are the cost savings, this is the environmental impacts, why are we not doing this, this is what we should be doing, is entirely reasonable at registrar and consultant level. But I think trickle down, because juniors are, the bandwidth of many juniors is so overwhelmed by stuff; they don't already know they're absorbing at an accelerated rate, but taking that on board as well is quite hard in a formal setting. - Participant 024

## Discussion

Barriers to green prescribing are multi-faceted and complex. Despite regular interaction with inhalers, very few participants would feel confident or consider switching a patient's inhalers from an MDI to a DPI whilst they were an inpatient. As evidenced by the initial audit data, only 2% of patients were identified as having undergone a switch during their inpatient stay. This is despite evidence showing equivalent efficacy of treatment between the two inhaler forms [21].

The largest barriers identified were a lack of knowledge about inhalers and awareness of more sustainable alternatives. Limited teaching at the medical school and postgraduate level and poor sharing of information around the sustainability of inhalers were also contributing factors. Even if a resident doctor has knowledge of inhalers available in their local formulary, there is an ever-expanding choice amongst inhaler devices and formulations, the availability of which varies between hospital trusts and regions. The current rotational nature of training pathways in foundation, core and specialty training means that resident doctors must continue to familiarise themselves with varying inhalers. This also makes the development of a national guideline around which inhaler device to use impossible.

These barriers had a clear knock-on impact on the perceived knowledge and confidence of participants in switching a patient's inhaler. One resident doctor mentioned that the 'bandwidth' of resident doctors is overwhelmed with information, implying the lack of time to increase their knowledge on sustainable medical practices. Ten participants who were invited for an interview declined due to time pressures, highlighting that sustainability discussions remain low on the prioritisation list of many resident doctors. A recurrent theme identified was the lack of clear guidelines and prompts when using e-prescribing. With an understanding of the increasing workload of resident doctors and pharmacists in the NHS, an easy-to-access flowchart and prompts would help improve compliance with switching patient inhalers and increase confidence to do so. The new BTS/NICE asthma guidelines published in November 2024 contain a decision aid; however, it is very limited in its recommendations about potential alternatives to MDIs [22].

There have been effective sustainable interventions within healthcare that have overcome these barriers. Examples such as the 'gloves off campaign' have made an impact on the carbon footprint of the NHS, saving 286 tonnes of carbon dioxide equivalent (CO_2_e) [23]. This successful campaign highlights how effective the dissemination of information can modify the behaviour of health professionals. Inhalers pose complexity through their combination of medications, as well as a plethora of devices to choose from; therefore, campaigns for prescribing SABA DPIs could be a simple starting point for intervention.

Effectively delivering information around green prescribing comes with its own difficulties. 'Mass emails' and 'busy posters' were deemed ineffective ways of influencing prescribers. Small group teaching, simulation and case-based teaching were commonly the preferred methods of teaching. Teaching from senior colleagues whilst on the ward was thought to be an effective and preferred method of learning. This highlights that targeting not only resident doctors and pharmacists but also senior colleagues may also have a positive impact.

Current literature

The current evidence supports that switching from MDIs to DPI does not impact asthma control whilst, as predicted, halving the associated carbon footprint [14]. This has been reiterated in systematic reviews [24,25] and within 'real-world studies' [26,27], alleviating the concern that switching to non-MDI inhalers would impact the clinical care or disease control of patients.

The current effort within and outside of healthcare is to reduce the carbon footprint of industries and individuals. Recent literature continues to highlight the significant impact inhalers can have on the environment. Hydrofluorocarbons present within inhalers have a global warming potential 1300 times higher than carbon dioxide alone [28]. An understanding of 'green inhalers' is key to reducing this GHG burden. From switching from MDI inhalers to DPI, there is potential for a significant reduction in the carbon footprint of the healthcare sector [29,30]. In a large Dutch-centred study, it was shown that switching to non-MDI inhalers saved 63 million kg of CO_2_e on average [31]. Furthermore, an example of switching from a combination of Seretide Evohaler and Ventolin Evohaler to Relvar Ellipta and Ventolin Accuhaler can save 422 kg CO_2_e a year [32]. That is a reduction in carbon emissions greater than an individual avoiding all food waste for a year (370 kg CO_2_e) and almost equal to switching from a petrol car to a hybrid car (500 kg CO_2_e) [33].

The use of SABAs contributes to 70% of inhaler-related emissions [34], with one Ventolin Evohaler contributing 205 kg CO_2_e annually [13]. Although there has been a shift within Europe of reducing the SABA burden, the United Kingdom remains an anomaly. The GHG burden from SABA use in the United Kingdom is treble that of other countries within Europe [35]. The new BTS guidelines published in November 2024 aim to reduce this burden with the use of AIR/MART therapies [36]. Although this decision was implemented based on clinical data from studies such as SABINA, its subsequent positive environmental impact cannot be understated [37]. The new guidelines also feature a decision aid called 'asthma inhalers and climate change' [22]. This is a step in the right direction; however, there is still room for clearer, more precise guidelines to further promote green prescribing.

In a struggling system such as the NHS, often, the environmental impact of its services is not a priority. Switching from MDIs to DPI has been shown to be a potential significant financial benefit alongside its positive environmental impact [27,38]. The prevention of hospital admissions is an effective way to reduce costs and lower the environmental impact of an individual on the healthcare system. It has been shown that patients with poorly controlled asthma have eight times more admissions than those who are well-controlled [15]. Furthermore, incorrect inhaler technique was shown to cost €782 million in a single year across Spain, Sweden and the United Kingdom in 2015 [39]. Many participants were hesitant to change a patient's inhalers. However, doing so whilst an inpatient could offer a great opportunity to ensure a patient's inhaler technique, therefore increasing compliance. The literature shows how important correct inhaler technique is in asthma and COPD control. Although switching to a DPI can be very effective at minimising the GHG burden of a patient's inhaler use, the literature suggests that correct inhaler technique is vital in patient compliance and exacerbations leading to hospital admissions [40,41]. This is an important factor in reducing the carbon footprint of the healthcare system, which should be considered alongside a drive to switching to greener inhalers.

Teaching on sustainability within the medical profession is changing. As of 2018, the GMC included a learning outcome for all medical school graduates, which states that they must 'outline the principles underlying the development of health, health service policy and clinical guidelines, including principles of health economics, equity and sustainable healthcare' [42]. As highlighted in this research, the effective teaching of medical students has raised a continued challenge. With sustainable healthcare being a vast-reaching topic, research has shown that the most effective method is to integrate it into all aspects of healthcare [43]. Incorporating it in this way allows the understanding and recognition of prevention as the most effective tool to succeed in reaching a more sustainable healthcare system. Although some notable positive steps have been taken forward in sustainable healthcare education [44-46], the 2020/2021 review highlighted that there is still a way to go [47].

Strengths and limitations

This qualitative analysis is the first to investigate the attitudes of secondary care physicians towards inhaler prescribing practices within the context of the NHS green agenda. It incorporates perspectives from resident doctors at various stages of training, alongside pharmacists with prescribing experience, to provide an initial analysis of this issue. Resident doctors, often at the forefront of clinical care, managing medical admissions and conducting ward rounds, play a central role as primary prescribers. Resident doctors are often fine-tuning patient medications with pharmacists' opinions. Furthermore, as the consultants of the future, their attitudes and practices offer valuable insights into the potential long-term influence of the identified barriers and facilitators in promoting sustainable prescribing practices.

The qualitative interviews were conducted by two resident doctors affiliated with the hospital trust, interviewing peers with whom they may have previously worked during medical admissions or ward rounds. This dynamic may have influenced participant responses, as participants might have refrained from expressing thoughts they perceived as inadequate in the presence of the interviewers. Furthermore, the participants received a preparatory leaflet prior to the interviews, which may have prompted them to undertake additional research on the topic to appear knowledgeable.

Additionally, pre-existing professional relationships could have introduced interviewer bias, potentially affecting the framing or delivery of questions. To mitigate such bias, the interviewers engaged in reflexive practice throughout the study. The research was conducted within a large teaching hospital featuring a dedicated respiratory department, potentially impacting participants' exposure to patients requiring inhaler use. A significant proportion of such patients may have been managed directly by the respiratory team, which could contribute to varying levels of knowledge and familiarity with inhaler prescribing compared to smaller hospitals, where such focused exposure might be less common.

The findings of this study are influenced by the experiences of individuals within the trust, which operates as a teaching hospital. Consequently, the postgraduate medical training and exposure to different inhalers amongst participants may differ from those in non-teaching hospitals, possibly yielding disparities in knowledge and clinical practice regarding inhalers. However, we felt that this is unlikely to pose a significant limitation due to the nature of postgraduate medical training in the United Kingdom; the participants interviewed in this study are likely to have had experience working in non-teaching hospitals in regions with differing inhaler formularies. There was a sustainability clinical fellow working within the respiratory department at the time, which may have influenced engagement in the project and responses around awareness.

Implications for future services and research

The findings of this project highlight the need to enhance the visibility of sustainable transformations within healthcare practices, along with medical education, enabling clinicians to make informed decisions regarding sustainability initiatives. It is essential to integrate sustainability directly into the medical team rather than relegating it to an independent body, as this approach will foster collaboration, idea generation and awareness.

Whilst this research specifically examines clinician attitudes towards environmentally sustainable inhaler prescribing, there remains a need for continued investigation into strategies for reducing the carbon footprint in asthma and COPD care. Such efforts might focus on improving disease control and reducing hospital visits and admissions.

As an outcome of this sequential mixed-methods study, a local inhaler guideline was developed to support resident doctors in their decision-making processes (Appendices).

## Conclusions

The NHS's use of inhalers contributes a sizeable portion of the carbon footprint of the United Kingdom. Switching to DPIs can help reduce this burden. However, multiple perceived barriers exist to prescribers making these switches in an acute hospital setting. These include knowledge, awareness and confidence. Guidelines and electronic prescribing prompts may offer a solution to help make positive changes to inhaler prescribing practices and reduce their carbon footprint.

## Figures and Tables

**Figure 1:**
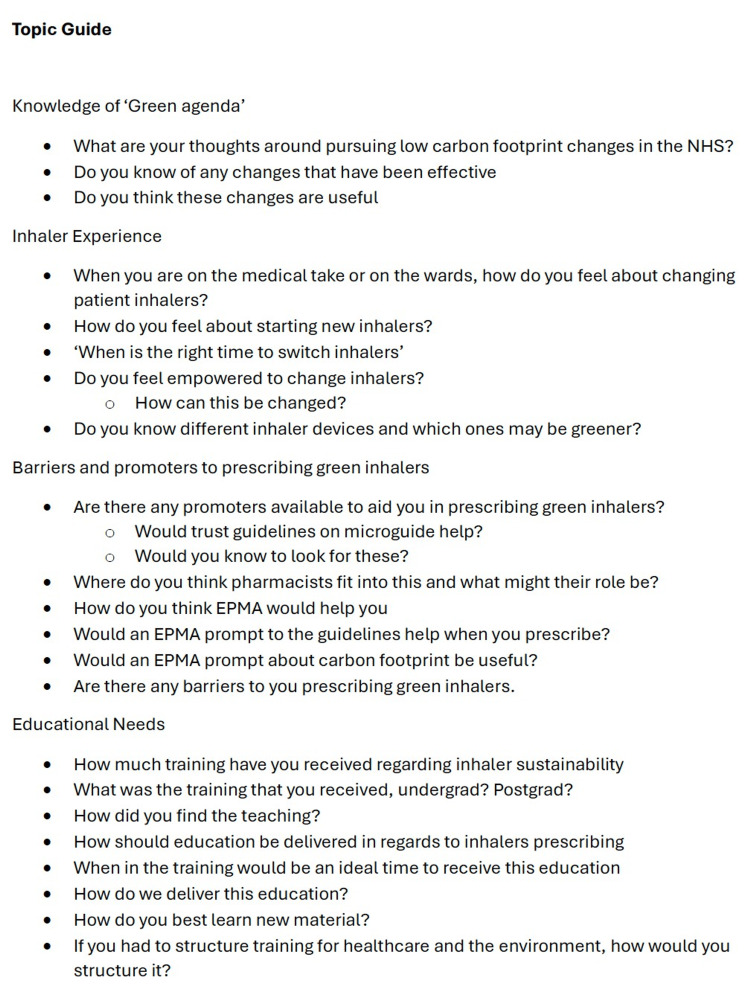
Topic guide for qualitative interviews

## Appendices

Figure 1 shows the topic guide.

Table 4 shows the codes and their descriptions.

**Table 4:** Table of codes and their descriptions MDT, multidisciplinary team; EPMA, Electronic Prescribing and Medicines Administration; SIM, simulation

Code	Code summary
Barrier to intervention	Barriers to switching inhalers include the lack of empowerment, time pressures, knowledge gaps, resistance from staff and cost
Prioritisation	Changing inhalers is a low priority, should be done on the ward and faces challenges like the lack of empowerment, communication issues, workload and prioritising patient outcomes over environmental impact
Opportunity	Junior doctors lack time to learn about inhalers due to the rotational nature, suggesting that exposure through conferences, trust intranet, EPMA prompts and pharmacist support, along with earlier involvement in sustainability, would enhance their confidence and knowledge
Modifying prescribing behaviour	EPMA alerts, guidance and pharmacist support would be useful in prompting inhaler switching and empowering junior doctors
MDT involvement	Pharmacists and specialist nurses are pivotal in helping with inhaler switches, advising on technique and decision-making, both on the ward and at discharge
Learning	Participants prefer small group, face-to-face and interactive teaching methods over disseminated bulletins and online teaching, with a preference for anecdotal, case-based learning and inputs from senior colleagues
Interventions	Raising awareness and educating on inhaler switches can be effectively achieved through teaching, posters and prescribing prompts
Inhaler prescribing behaviour	Poor knowledge causing low confidence and familiarity with prescribing inhalers causing reliance on seniors, with prompts and better education potentially improving confidence
Inhaler knowledge	Poor knowledge, exposure and understanding of inhalers and their carbon footprint leading to low confidence in prescribing
Inhaler education	Participants have a lack of prior knowledge and exposure to inhalers
Inhaler confidence	The lack of confidence due to knowledge in switching inhaler devices
Impact of green intervention	Unaware of trust policies, some impact felt of green interventions, particularly anaesthetic gases. Participants feel the lack of drive from the trusts towards green agenda
Health policy	The lack of awareness and culture around the NHS green agenda due to communication
Healthcare culture	Participants feel that the current healthcare culture does not promote the green agenda. Concerns around cost and patient flow
Guidelines	A lack of specific green inhaler guidelines. Local and trust policies would aid decision-making and improve confidence
Green inhaler awareness	Some interest and awareness around green inhalers but barriers like the lack of knowledge and recycling. Some feel it is a primary care issue
Green awareness initiative	Poor level of awareness of green initiatives locally and nationally
Green agenda awareness	Varying levels of awareness about the NHS green agenda, with some understanding of carbon footprint reduction and sustainability efforts. The focus is on cost-saving, and there is a lack of broad-scale awareness and communication
Education modality	Interactive, nonuniform methods such as small group teaching, in-person coaching/mentoring and interactive sessions preferred. Feel that online teaching and posters are inadequate
Education delivery	Small group in-person teaching at multiple levels, posters for awareness and interactive learning methods such as SIM would be most effective for education on inhaler switching
Changing inhalers	The lack of knowledge to change inhalers, which lowers confidence. Guidance and senior decision-making would improve confidence. Felt that in the acute setting switching, inhalers would be inappropriate
Education about the green agenda	Participants have received little to no sustainability education, with most training focused on inhalers at medical school or obtained through personal interest

University Hospitals Sussex inhaler switching guidelines

The University Hospitals Sussex inhaler switching guidelines can be found in the following: https://drive.google.com/file/d/1BzeC0kOz0oa-3ZCKtiJ_xXXQMyX1eMY9/view?usp=sharing.
